# Supplementary material for: Are carbon emissions trading and green financial instruments synergistic? -Comprehensive quantitative research based on content analysis
Source: PLoS One. 2024 Mar 7;19(3):e0298601. doi: 10.1371/journal.pone.0298601 (PMC10919601; doi:10.1371/journal.pone.0298601)
Supplement: S1 Data — (ZIP) [file pone.0298601.s001.zip › Data/地级市Z_F工作报告.zip!/║┌┴·╜¡/╞δ╞δ╣■╢√╩╨/└┤╫╘╣1⁄2╓┌║┼í╛┬φ┐╦╩2╛▌═°í┐_2009.docx]

各位代表：

　　现在，我代表市人民政府向大会报告工作，请予审议，并请各位政协委员提出意见。

　　一、2008年工作回顾

　　2008年是不平凡的一年。我们以科学发展观为指导，在省委、省政府和市委的正确领导下，按照市委“十二个坚持”的要求，团结带领全市广大干部群众，积极应对错综复杂的宏观经济环境变化，努力克服前进道路上的诸多困难，圆满完成了市十四届人大二次会议确定的各项任务，国民经济和社会发展取得显著成绩。完成地区生产总值665.9亿元，增长14.8%。财政总收入59.2亿元，为预算的115%，增长28.7%；地方财政收入36.4亿元，为预算的116.3%，增长30.2%。财政总支出123亿元（包括上级财政补助支出），增长21.6%；地方财政支出86.2亿元，为预算的114.6%，增长20.5%。城镇固定资产投资209亿元，增长31.4%。续建和新开工5000万元以上项目54项，实际完成投资76.6亿元，为年计划的123.3%。17项列入省重点推进的项目，已有13项开工建设，完成投资28.2亿元。抢抓国家扩大内需政策机遇，43个项目获得批准，总投资35.7亿元，其中国家投资4.8亿元。

　　（一）农村经济全面发展。积极推进“四个换位”，大力发展现代农业，着力搞好农产品的产加销，促进农业增产、农民增收。粮豆薯总产153.9亿斤，农村居民人均纯收入4031元，均创历史最高水平。通过及时发布粮食供求信息、加强粮食供销协作、支持农产品加工企业满负荷生产、争取中储粮指标和进关车皮等措施，有效缓解了春夏卖粮难问题。畜牧业稳步发展，实现产值106.9亿元，增长13%。农业产业化步伐加快，引进和启动24个千万元以上的农产品加工项目，规模以上龙头企业达185家。农业生产条件得到改善，治理水土流失46万亩、草原“三化”57万亩，新增旱涝保收田56万亩。编制了全市造林绿化规划，植树造林30.2万亩。积极推进北引扩建、泰来抗旱灌溉引水工程等重点水利设施建设，完成了三大灌区续建配套、黑土区治理及水库消险加固年度建设任务，投入农田基础设施建设资金2.5亿元，增长68%。新建农机合作社51个，田间作业综合机械化率达82%。累计转移农村劳动力93.6万人，实现劳务收入48亿元，增长18.5%。发放综合直补、粮食、生猪、良种等补贴资金17.6亿元。投入新农村建设试点村帮建资金2572万元，第一批试点的127个村全部通过省验收。162个贫困村整体脱贫，农村贫困人口下降11.1%。农村综合改革不断深化，为农服务代理制全面推进。流转土地943万亩，实现规模经营583万亩。集体林权制度改革试点工作如期完成，场县共建取得积极进展，户籍管理制度改革率先在全省全面实施。村级主要负责人经济责任审计取得良好效果。我市荣获第二次农业普查全国先进单位。

　　（二）工业经济平稳较快增长。坚持走新型工业化道路，加大服务支持企业力度，增强工业经济实力和发展后劲。规模以上工业主营业务收入591.2亿元、税金30.3亿元，分别增长30.7%和20.5%。落实企业国家和省各类资金和贴息3.64亿元。重奖贡献突出的企业及个人482万元，对一重、齐重数控、齐二机床等企业在土地使用、退休人员医保待遇、企办学校职工接收上给予支持，协调解决了企业的原料和运力不足等问题。齐重数控与浙江天马股份公司、齐二机床与中国通用技术有限公司实现战略重组。完成8户国企改制、10户国企政策性破产工作。续建和新开工一重国际一流铸锻钢基地等15个亿元以上项目，总投资108亿元，完成投资31亿元；实现齐轨道装备公司总体改造等14个5000万元以上项目竣工投产。完成了北钢500万吨特钢项目规划，腾翔30万吨铸锻件项目进展顺利。争取省级以上科技创新项目33项、资金3564万元。2个项目列为国家科技支撑计划重大项目，一重集团15000吨水压机获国家科技进步一等奖，4个项目获省科技进步一等奖，新晋升6个省级以上重点实验室、企业技术中心，实施100个技术创新项目，10户企业晋升为国家高新技术企业。实现高新技术产业产值230亿元、利税18亿元，分别增长28%和78.2%。列入松花江水流域污染防治规划的项目全部开工建设，完成投资3.62亿元。市中心城区污水处理厂日处理能力由3万吨提高到8万吨。关停了纳入国家淘汰落后产能名单的5户企业。万元GDP综合能耗降低7.05%，削减化学需氧量7605吨、二氧化硫排放量2680吨。

　　（三）第三产业不断壮大。突出发展以旅游业为重点的第三产业，努力促进经济结构优化升级。出台了加快发展旅游业的若干意见，积极推进旅游支柱产业发展。实现旅游收入40亿元，创汇611万美元，分别增长23.3%和30.2%。按照把扎龙景区建成5A级世界著名景区、尼尔基斯湖景区建成国家级冰雪和水上大众运动基地的目标，聘请中国规划设计研究院进行规划设计，尼尔基斯湖国家大众滑雪基地已投入使用。按照三年叫响关东文化旅游节的目标，进一步突出“关东”特色，丰富活动内容，提升了关东文化旅游节的层次和水平。先后赴温州、厦门等地举办旅游推介活动13场次，进一步提高了我市旅游产品的影响力和吸引力。第八届绿博会、第五届小交会等会展活动成效显著，经贸成交总额114亿元，增长16％。积极促进服务外包业发展，引进亿阳集团佰科信通公司建立IT人才培训中心和软件检测中心。进一步繁荣商贸流通业，新玛特休闲购物广场竣工营业，百货大楼扩建工程投入使用。

　　（四）对外开放取得新成效。积极走出去、引进来，广泛寻求支持合作，努力扩大对外开放。坚持以企业为主体、项目为纽带的务实招商原则，通过赴天津、上海、杭州等地招商，参加哈洽会，举办中国光彩事业鹤城行等活动，落实招商引资项目1357项，到位资金204.2亿元，增长39.6%。其中，直接利用外资5960万美元，增长22%。新引进亿元以上项目36项，到位资金48.7亿元。对外经贸不断扩大，完成进出口总额5.82亿美元，增长116.4%，增速位居全省第一。其中，出口5.1亿美元，增长131.6%；进口0.72亿美元，增长48%。按照省建设哈大齐工业走廊核心园区和重点园区要求，科学规划建设重点园区和铸造、机加等园区，协议入区企业262户，总投资150亿元；实际入区企业132户，总投资116亿元，完成投资72亿元。腾翔铸锻造基地、农业高科技示范区、北大仓物流园区等项目落户鹤城科技产业园区。积极争取各方面支持，相继走访和接待了17个中央部委和单位、124个大型企业集团。出访了韩国、日本友好城市。与天津滨海新区、上海徐汇区、温州市建立了友好关系，与天津市签订了农产品战略合作协议和建设内陆无水港合作协议。

　　积极搞好对航天英雄翟志刚和英雄航天员刘伯明的宣传活动。制作了《活力鹤城？明信片》，系统展示了我市的深厚文化底蕴和独特发展优势。拍摄了在国内外发行的大型文献纪录影片《鹤舞北疆》，集中反映了我市英雄城市的历史、老工业基地的巨大贡献和改革开放以来的奋斗历程。

　　（五）城乡建设步伐加快。积极开辟城乡建设新的发展空间，拓宽新的资金来源渠道，实施新的重点工程。高起点、高水准编制了劳动湖水系改造、公铁联运客运枢纽站、沿江发展、新农村建设等专项规划，哈齐铁路客运专线齐齐哈尔段和公铁联运客运枢纽站建设已顺利启动。新建和改造城区道路12万平方米，升级改造了卜奎北大街北段、新江路等6条重点路段。完成了城区热网A区二期工程，并网供热620万平方米，7.5万户居民受益。天然气复线工程昂昂溪至富拉尔基和中心城区两条支线建成投产，年供气能力达到6.5亿立方米，提高12倍。新建鑫海家园、新合家园等11个住宅小区，建筑面积124.7万平方米，增长15%。为1.4万户、3.2万人发放廉租住房补贴2100万元。成功争取齐富大桥及高等级公路建设项目。完成高速公路建设征地518万平方米；新建农村公路3861公里，为年计划的107.6%，实现通乡公路全硬化。城市重点建设项目发行企业债券15亿元工作进展顺利。与省开行签订了城市棚户区改造项目贷款10亿元协议，已到位资金4亿元。拆除违章建筑410处、2977平方米，加大牌匾广告、乱贴乱画治理和垃圾废物清理力度，市容市貌有了新改观。地理信息公共服务平台已通过省测绘局鉴定。编制了城区绿地规划，制定了城区绿化方案。城区栽花铺草植绿篱100万平方米、植树30.2万株，绿化覆盖率达31.1%，人均公共绿地面积7.1平方米。

　　（六）社会事业全面进步。大力发展教育、文化、卫生、体育等社会事业，统筹经济社会发展。新增教育建设项目21个、建筑面积7.39万平方米。中小学生辍学率分别低于国家规定标准0.96和1.7个百分点，高中阶段毛入学率达53.1%，义务教育阶段的农民工子女全部在校就读。职业学校招生2.09万人，毕业生就业率达95％。克山、克东被国家和省确定为特殊教育金钥匙工程实验县。齐齐哈尔大学进入全省10所高水平建设大学行列，齐齐哈尔医学院迁入新址。市级重点学科带头人梯队新增9个，26名专家享受省、市政府特殊津贴，6个项目获市长特别奖。话剧《风刮卜奎》荣获曹禺戏剧剧本奖，入围国家舞台艺术精品工程奖。市马戏团赴日本、美国等7个国家演出900余场。群众业余文化生活丰富活跃，文化市场规范有序。率先在全省免费开放市博物馆，金长城资源调查进展顺利，对4个重点文物保护单位进行了修缮，对191处历史文化建筑实施挂牌保护。新建乡镇文化站7个，市图书馆新馆主体工程建设完成。实现了中心城区电视传播数字化。进一步整合了城市医疗卫生资源。新建社区卫生服务中心、服务站69个。争取国债建设资金2921万元，加强了乡镇卫生院建设。人口自然增长率为2.11‰，生殖健康援助行动延伸到乡、村两级。成功承办了第十一届全国冬运会，受到国务院、国家体育总局表扬，我市体育代表团实现了金牌总数、团体总分第三的历史性突破。冰上体育设施综合水平达到亚洲第一。圆满完成了北京奥运会和世界大学生冬运会火炬传递任务。我市残疾人运动员在北京残奥会上获得了两金、一银、一铜的优异成绩。

　　（七）人民生活明显改善。把提高人民群众生活质量作为政府工作的出发点和落脚点，千方百计改善民生。城镇居民人均可支配收入11028元，增长15.3%。居民储蓄存款余额451亿元，增长23%。社会消费品零售总额257.3亿元，增长22.3%。居民消费价格指数累计上涨4.85个百分点，低于全省平均水平1个百分点。与人民群众生产生活密切相关的54件大事实事基本完成。扎实推进低保提标和社保、医保扩面工作，发放城市低保金2.6亿元、月人均补差95元，发放农村低保金7208万元、年人均补差564元，实现了应保尽保。纳入城镇养老保险44.1万人、城镇居民医疗保险46万人。争取国家和省补贴8932万元，解决了符合国家政策性破产规定的27户国有企业、26601名退休人员医保待遇问题。新型农村合作医疗参合率达93%，累计筹资1.6亿元，发放补助款9593万元。深入实施创业带就业工程，发放小额贴息贷款3.6亿元，继续保持全省第一、全国领先。新增就业11.8万人，零就业家庭月报告期内动态为零。全面完成企业历史拖欠职工工资26.47亿元的解欠任务，争取国家补助14.4亿元，做到了公开公正、应补尽补。拨付3.1亿元（其中市财政5936万元），全部解决了中省市直企业企办学校1万多名教职工接收问题。规范和调整了市区机关事业单位职工津贴补贴，提高了环卫工人工资。全力推进城市棚户区和农村泥草房改造“天字号”工程，完成棚改拆迁43万平方米，回迁安居房建设100栋、50万平方米，6427户居民住上了回迁安置楼。城区人均住房面积23.2平方米，增加0.6平方米。完成农村泥草房改造4.5万户、340万平方米，农村住房砖瓦化率提高3.7个百分点。积极搞好热源整合和燃煤供应，保证了群众温暖过冬。新打人饮井168眼，解决了农村8.4万人饮水安全问题。我市被人民网评为“市民最满意城市”。

　　（八）社会管理进一步强化。认真履行社会管理职责，努力营造安定有序的社会环境。安全生产形势总体稳定，各类事故死亡人数下降18.5%。制订了应急处置总体预案及42个专项应急预案，有效处置了“6.5”光气泄漏、“7.7”地震、东兴农机非法集资、“12.24”供暖故障等重大突发事件。深入开展“秋风行动”，有力打击了刑事犯罪，社会治安持续稳定。解决了公检法司系统机构编制问题，拨款1000多万元用于反恐设施、公安“三基工程”建设。“五五普法”教育和人民调处、法律援助工作取得实效。加强食品药品监督管理，全面清理整顿问题奶粉，出台生鲜乳购销管理规定，有效防止了危害群众生命健康安全的事件发生。深入开展矛盾纠纷排查化解、重信重访专项治理，对重大信访案件实行领导包案制度，下大力气处理17个历史遗留的重大信访问题。列为市政府督办事项的群众来信来访全部办结。

　　（九）政府自身建设得到加强。紧紧依靠市委的领导和市人大、市政协的监督，重大事项、重要工作及时向市委请示报告，依法向市人大报告工作，定期向市政协通报情况。办理人大代表议案78件、政协委员提案372件，办结率均达100％。起草、审修地方性法规（草案）2部、政府规章9件。在政府决策中发扬民主，科学论证，集体决定，努力营造团结和谐的工作环境。加强公务员队伍建设，严格要求，严格管理。坚持依法行政，推进政务公开，制定了市政府提交市委常委会议审议事项规则、市政府工作规则、编制委员会规则和公务员调动、大额资金和市长预备费使用、土地市场管理办法等制度规定，从源头上预防了腐败现象发生。

　　民族宗教、机关事务、新闻出版、档案、地方志、人防、气象、妇女儿童、民兵预备役、双拥优抚等工作取得了新成绩。

　　在过去一年的政府工作中，我们始终坚持把发展融入国内外大局，密切关注宏观环境变化及趋势，及早制订应对突发事件的预案，未雨绸缪，妥善处置，努力化解各种不利因素；坚持突出重点，把握全局，打牢基础，谋划长远，对事关经济社会发展的重大项目、重要工作，统筹考虑，合力推进，务求实效；坚持科学理政、依法行政、从严治政、为民执政，发扬“5+2”、“白+黑”的敬业创业精神，抢抓机遇，深化改革开放，积极争取各方面支持，创造性开展工作。对于这些行之有效的做法，我们在今后工作中，一定继续坚持和完善。

　　各位代表，上述成绩的取得，是省委、省政府和市委正确领导的结果，是市人大、市政协监督支持的结果，是全市人民共同奋斗的结果。在此，我代表市人民政府，向全市各族人民、各民主党派、各人民团体和各界人士，向所有关心、支持我市改革发展的同志们、朋友们，表示衷心的感谢！

　　我们也清醒地认识到，全市经济社会发展和政府工作中还存在一些困难和问题。在经济发展方面，经济增长主要靠传统产业拉动，新兴产业比重较低；对经济发展牵动力强的大项目还不多；运用金融和资本市场手段破解资金难题的本领还不娴熟；国际金融危机等宏观经济环境变化，已给部分企业生产经营带来了影响；农业基础设施薄弱，抵御自然灾害和市场风险能力不强，卖粮难问题尚未完全破解；对市场和流通体系的培育和建设还需进一步加强。在社会发展方面，部分群众收入偏低，生产生活条件有待改善；就业形势严峻，大学毕业生和“4050”人员就业难问题比较突出。在政府自身建设方面，少数公务人员责任意识不强，工作本领和效率不高，不正之风和腐败现象还不同程度地存在。对于这些问题，我们在今后工作中，一定高度重视，认真加以解决。

　　二、2009年工作任务今年是新中国成立60周年，也是推进“十一五”规划顺利实施的关键一年。今年的政府工作，既面临着国际金融危机的影响呈继续加深趋势、社会矛盾更加突出的严峻考验，也面临着国家扩大内需、加大投资、促进经济平稳较快增长的难得机遇。今年政府工作的总体思路是：以党的十七大和十七届三中全会精神为指针，深入贯彻落实科学发展观，按照市委既定的发展定位、发展方向和发展思路，积极应对宏观经济形势变化，把保持经济持续较快增长作为首要任务，抢抓发展机遇，转变发展方式，优化发展结构，改善发展环境，加快新型工业化进程、新农村建设和第三产业升级，深化改革开放，着力保障和改善民生，推动经济社会又好又快、更好更快发展。

　　今年全市经济社会发展的主要预期目标是：地区生产总值增长12%，力争14%以上；地方财政收入增长12%，力争15%以上；城镇固定资产投资增长30%，力争40%以上；规模以上工业增加值增长13%，力争18%以上；高新技术产业产值增长26%以上；社会消费品零售总额增长15%，力争20%以上；进出口总额增长15%，力争18%以上；城镇居民人均可支配收入增长8%，力争10%以上；农村居民人均纯收入增长6%，力争8%以上；居民消费价格总水平涨幅控制在3%左右，城镇登记失业率控制在4.3%以内，万元生产总值综合能耗下降6%以上。这些目标，既考虑了我市经济发展的潜力和金融危机的影响，又遵循了科学发展、快速发展、和谐发展的要求，力求实现速度与质量相统一，经济发展与社会发展相协调。

　　实现今年经济社会发展目标，需要坚持的原则是：全面把握宏观经济形势，趋利避害，把不利影响降到最低，把有利条件用到最好；抓住国家扩大内需、加大投资的机遇，以大项目建设为依托，力促经济社会加快发展；深化改革，扩大开放，推进制度建设和创新，增强发展活力；更加注重社会建设，以人为本，改善民生。当前，我们要全面贯彻落实中央和省应对国际金融危机、扩大内需、保持经济平稳较快增长的各项政策措施，把项目建设摆上更加突出的位置，作为实现经济社会又好又快发展的首要任务和根本举措，举全市之力进行谋划、推进和落实。初步安排，实施和谋划先进制造业、现代农业、能源交通、节能减排、城市建设、经贸流通和社会事业等方面90个重点项目，总投资734.3亿元，当年完成投资92.1亿元。把确保新的经济增长点高增长、快增长，作为应对危机保增长的关键措施，突出培育和发展高新技术、旅游、内外贸、房地产、医药、建材建筑、林木、矿产资源开发、节能环保、会展、物流、信息服务外包、文化、体育等14个产业，努力创造经济发展的新优势。

　　（一）积极推进农村改革发展。高度重视“三农”工作，以“四个换位”为统领，以建设现代农业为重点，努力促进农业增效、农民增收和农村繁荣。提高粮食综合生产能力。加快省千亿斤粮食产能工程和松嫩平原农业综合开发试验区建设，调整种植业结构，大力推广农业先进实用技术，搞好2000万亩优质粮食产业带建设。绿色食品种植面积达到842万亩。大力推进“主辅换位”。优化畜牧养殖结构，推进良种工程，加强草原改良，搞好疫病防治，实行科学饲养，扩大奶牛、肉牛、生猪、肉羊、肉鸡养殖规模，畜牧业产值增长7%以上。加快生态型养殖基地建设，绿色畜禽养殖总量增长5%以上。深入推进产业化经营。壮大龙头企业规模，立足资源优势培育一批、整合一批、招引一批项目，提高农产品精深加工比重，力争食品工业增加值增长29%。完善基地与龙头企业利益联接机制，发展订单农业，推动基地专业化、标准化、规模化发展。实施品牌战略，加大品牌整合力度。加快“一村一品”工程建设。高度重视农产品质量安全。认真落实《农产品质量安全法》，推广农业标准化生产，加强农产品质量安全认证和行政执法。大力发展劳务经济。深入开展阳光工程培训，提高农民转移就业本领。积极开发劳务市场，改善输出结构，引导农民有序转移。完善服务体系建设，提高劳务输出组织化程度。积极扶持农民工返乡创业就业。加强农村基础建设。利用北欧长期贷款加快组建现代农机专业合作社，推动大马力拖拉机及其配套农具建设。加大水田开发力度，新增水田40万亩。继续抓好三大灌区节水改造、末端渠系及骨干工程配套建设，启动尼尔基水库下游引嫩一期工程扩建及泰来抗旱灌溉引水工程内部配套建设，扩大旱涝保收田面积。推进防汛抗旱减灾体系建设，搞好水库消险加固工程，力争齐富及嫩干堤防达标工程立项，完成城区堤防达标工程建设。解决农村10万人饮水安全问题。抓好水土保持和生态建设，新增4个黑土区治理重点县。加快发展路、水、电、房、医、教、能、保等农村公共事业。进一步搞好扶贫开发，力争实现4.7万名农村居民脱贫。深化农村各项改革。把土地适度规模经营改革作为农村改革的主攻方向，坚持依法、自愿、有偿原则，认真执行“三不规定”，因地制宜选择适度规模经营模式。全市新增土地适度规模经营面积达到耕地总面积的10%以上。全面完成集体林权制度改革。深化农村金融改革，做好依安村镇银行试点工作。加强粮食流通体系建设，发挥国有粮库作用，抓好政策性粮食收购、经营性粮食购销，突出解决卖粮难问题。我们这个农业大市，就是要建设一个农业基础更加牢固，产、加、销更趋协调发展，绿色食品产业规模更大，县域经济实力更强，广大农民收入更多，城乡一体化进程更快，繁荣、富庶、文明的社会主义新农村。

　　（二）促进工业结构优化升级。以大项目建设为重点，全力为企业搞好服务，提升工业发展水平，加快新型工业化进程。支持企业自主创新。深入落实《科学技术进步法》，发挥财政资金的导向作用，支持企业加大研发、技改投入，鼓励发明创造，促进科技成果转化，增强自主创新能力。扶持企业创建国家级、省级企业技术中心、工程中心和重点实验室。开发新产品60种，实施重点技术改造80项。积极组织申报国家、省重大科技专项，提升重点产业发展水平。制定优惠政策，鼓励生物工程、新材料等产业加快发展，积极引进培育发展高新技术企业。继续支持企业争名牌创品牌。推动大项目建设。重点抓好一重铸锻钢基地、大型石化容器及核电主设备国产化、能源装备检测中心，北钢500万吨特钢，齐重数控高档数控机床产业化基地，齐二机床高档数控机床产业化、重型数控多工位压力机产业化，齐轨道装备公司产业升级、博天糖业100万吨甜菜加工、鸿源油脂30万吨大豆精深加工、昱诚公司30万吨氨基酸生物有机肥等25个项目建设。争取滚动实施3000万元以上工业项目35项。认真抓好节能减排工作。进一步落实节能减排综合性工作方案，严格实行考评制、问责制和一票否决制。加强对投资项目的环境评价和重点耗能企业的监管，淘汰落后产能，推广应用节约能源资源的新技术、新设备和新材料。抓好重点节能减排工程建设，全力推进松花江流域水污染防治规划项目实施。力争节能环保产业产值增长15%以上。切实为企业搞好服务。认真落实国家、省、市支持企业发展的政策措施，在立项、资金、运输、用地、社会保障、分离企业办社会等方面予以全力支持。畅通政企联系渠道，为企业提供有效快捷的信息服务。积极为企业搭建招商合作平台。继续推进企业改革。对具备条件、尚未改制的国有企业，因企制宜、分类指导，成熟一户、改制一户。抓好高负债、关停企业的依法破产，减负卸债，退出国有。支持国有企业并购重组，完善法人治理结构。多渠道筹措资金，继续搞好厂办大集体改革。加强国有资产监管，确保保值增值。大力发展非公有制经济。认真落实国家和省市促进个体私营经济发展的政策措施，积极服务、保障、支持非公有制经济发展，努力培育壮大一批信誉优、前景好的民营企业。引导组织创业者和就业者，适于干什么就干什么，能发展多好就发展多好，形成千家万户竞相发展、社会财富充分涌流的生动局面。力争非公有制经济增加值增长15%以上。我们这个老工业城市，就是要建设一个以市场为导向，以科技创新为引领，以大项目为支撑，结构更优，质量更好，总量更大，竞争力更强，在国内外具有重要影响的现代化装备工业基地。

　　（三）提高第三产业发展水平。着力壮大以旅游业为重点的第三产业，增强对经济发展的拉动作用。促进旅游业快发展大发展。全面落实加快发展旅游业的若干意见。按照“保护自然、展现质朴”的原则，加快编制全市旅游总体规划和专项规划，按新规划启动扎龙景区建设，完善尼尔基斯湖滑雪场配套设施，启动夏季水上运动项目规划、设计与建设。推进昂昂溪俄罗斯风情小镇、齐齐哈尔国家森林公园、金长城遗址公园、铁农园艺园等重点景区建设。依托历史、冰雪、温泉、鹤文化等特色优势资源，丰富旅游产品，延伸产业链条。理顺主要景区管理体制，加大吃、住、行等配套设施建设力度，加强从业人员队伍建设，提高接待服务水平。加强区域旅游合作，精心策划、开展旅游推介活动，开拓旅游客源市场。力争旅游总收入增长20%以上。壮大会展经济。高质量办好绿博会、小交会、观鹤节、和平节、关东文化旅游节等重要会节，广泛聚集人流、物流、资金流和信息流。加强与知名会展组织合作，积极承办各种展会，提高展会质量。力争会展业经贸成交额增长20%以上。发展信息服务外包业。抓紧制定加快发展服务外包业实施意见，搞好服务外包园区建设，服务支持亿阳集团佰科信通公司投资建设IT人才培训中心和软件检测中心。大力发展软件开发产业，扩大电子信息产品规模。扶持企业加大对传统电子产品的技术改造力度，促进信息产业优化升级。力争信息服务外包业销售收入增长15%以上。培育壮大物流业。重点围绕农产品、工业产品和原材料，引进培育发展物流配送企业。支持北大仓国际物流中心等企业加快建设。力争发展1-2家年营业收入超5000万元的物流龙头企业。繁荣商贸流通业。加快流通市场体系建设，优化商业网点布局，促进百花集团小商品商务中心等专业批发市场发展。扎实推进“万村千乡”市场工程，落实好“家电下乡”财政补贴政策，引导促进农村消费。强化城乡市场监管，规范市场经济秩序。我们这个旅游资源丰富、区位优势明显的城市，就是要建设一个“世界大湿地、中国鹤家乡”品牌更加响亮，现代服务业更加繁荣，东北地区重要的商贸物流中心和全国闻名的生态旅游胜地。

　　（四）扩大对外开放。拓展对外开放的广度和深度，大力发展外向型经济。扩大对外联系和宣传。进一步加强与知名企业、商会、财团和友好城市的沟通联系，扩大政府间、企业间和民间的交流合作。以《活力鹤城？明信片》和大型文献纪录影片《鹤舞北疆》为载体，扩大对外宣传，树立城市良好形象。开展务实招商。创新招商方式，突出政府的协调服务功能，组织好以企业为主体的招商活动。抓住世界性产业结构调整机遇，围绕国内外500强企业开展招商推介，主动承接引进一批牵动力强的项目。抓好签约项目的跟踪服务，提高履约率，确保早日建成达效。不断充实完善招商引资项目库。抓好园区建设。整合园区功能，加快基础设施建设，提高园区承载能力。加快哈大齐工业走廊齐齐哈尔规划区建设，重点抓好高新技术产业开发区、鹤城科技产业园区重大项目、富拉尔基开发区企业公寓二期工程建设。建立税收返还机制，引导县（市）区引资项目向示范区集中摆放，促进产业集群化、集约化发展。保持对外经贸稳定增长。适应国际市场需求变化，调整出口产品结构，加大对机电产品、高新技术产品和绿色农产品出口的支持力度。推动出口企业自主创新，扩大高附加值产品出口。扶持壮大国有骨干进出口企业，积极培育外向型民营企业，发展外商投资出口企业，形成多元化的外贸格局。鼓励企业开展以森林采伐、木材加工、建材生产、农产品种植加工等为重点的境外投资合作，大力发展以对俄为主的工程承包和劳务输出，不断拓宽合作领域。继续巩固与俄罗斯、韩国、日本等主要贸易伙伴关系，开拓中东、拉美、非洲等新兴市场，促进外贸稳步增长。我们这个内陆城市，就是要努力建设一个对外联系更加广泛，战略投资者更加集聚，对外经贸更加活跃，经济社会发展外向度更高的开放型城市。

　　（五）加快城乡建设步伐。大力推进城乡基础设施和生态建设，完善城乡功能，改善人居环境。突出规划引领作用。立足长远发展，着手搞好嫩江西岸鹤城新区规划，做好开发建设的前期准备。实施劳动湖水系改造规划，加快水系综合治理，与嫩江南北贯通，提高城市品位和发展活力。实施沿江发展规划，高质量建设沿江风光带。加快公铁联运客运枢纽站建设，以新站建设为契机，启动南部城区规划、设计与建设，建设以高新技术产业、教育文化卫生、商贸交通民居为支撑，集中国民族传统文化元素与现代服务功能于一体的现代化新城区。加快城乡基础设施建设。围绕城区重点规划区域建设，完成卜奎大街、曙光大街、青云街、水师路、西园路等主次干道升级改造，完善公共交通服务。推动西南城区热源项目建设。加快城区A区热网三期工程建设，力争新增并网供热1000万平方米。进一步加快小城镇、中心村、试点村、城中村建设。全面开工建设齐富公路及境内5条高速公路，新建农村公路2500公里。促进房地产业有序健康发展。全面落实国家促进房地产市场健康发展的政策措施，调整商品房供应结构，增加中低价位、中小套型普通商品房供应，建设商品房50万平方米，力争70万平方米。房地产投资增长30%以上。加强生态环境建设。加快实施全市三年造林绿化规划和城区绿化规划，重点抓好风沙口治理、江河绿化、城镇绿化、村屯绿化、农田草牧场防护林、公路绿化等重点工程建设。积极争取专项资金，综合整治龙、甘、泰等重点区域的生态环境，保护扎龙湿地，维护生态安全。植树造林27.2万亩。搞好35个绿地公园游园建设规划，启动秀水公园、马恒昌公园、新合公园等15个公园游园的景观建设。抓好卜奎大街绿化升级改造。城区绿化覆盖率达到34%。提高城市生活垃圾无害化处理率，推进村镇环境综合整治。提高城市管理水平。进一步规范土地市场化运营，提高土地利用效率和经济效益。继续推进城乡环境卫生管理由主干道向次干道、由商业区向居民区、由县级镇向建制镇延伸。规范路边经营，加大对违法违章建筑、牌匾广告、乱贴乱画的整治力度。严格物业管理市场准入制度，依法规范物业公司权利和义务，建立业主自治与物业企业管理相结合的管理机制。推广应用地理信息公共服务平台，推动城市管理数字化。我们这个北纬47°线以北的百万人口城市，就是要建设一个生态特色更加突出，文化特征更加鲜明，城市功能更加完备，人居环境更加优越的独具特色的现代化城市。

　　（六）做好财税金融工作。坚持依法理财治税，加快公共财政体制建设，促进经济与金融和谐发展。提高公共财政保障能力。重点推进26个财源项目建设，增强财政实力。继续完善部门预算编制改革，完成区级国库集中支付制度改革和非税直缴改革扩面工作，启动公务卡管理制度改革。规范非税收入管理，严格执行“收支两条线”。调整优化支出结构，积极争取资金，加大对民生工程、基础设施、生态环境建设的投入，严格控制一般性支出。加强税收征管。大力培育税源和新的税收增长点。落实好各项税收优惠政策，做好增值税转型工作。强化对重点税源的监控，进一步加大税务稽查和欠税清缴力度，堵塞税收征管漏洞，确保应征尽收。强化金融支撑。全面落实国家和省市关于金融促进经济发展的政策措施，切实加大对重点行业和重点项目的信贷支持力度。用好国家开发银行17亿元的授信额度。进一步完善中小企业担保体系，通过资本注入、风险补偿等方式，加强对信用担保公司的支持。鼓励金融机构建立专门为中小企业提供信贷服务的部门。拓宽企业融资渠道，加快一重、齐轨道装备公司等企业上市融资步伐。提高城投公司资本运营能力，做好发行15亿元企业债券工作，解决城市建设资金紧张问题。认真落实市委、市政府关于支持银行业发展的意见，建立有效的激励机制，搭建银企合作平台，帮助银行回收贷款，鼓励银行增加贷款投放，对贡献大的银行和个人给予奖励。我们就是要努力建设一个骨干财源更加壮大，新兴财源更快发展，金融支撑更加有力，经济综合实力更强的城市。

　　（七）大力发展各项社会事业。加强社会建设，完善公共服务，促进经济社会协调发展。全面发展教育事业。落实义务教育经费保障政策，使经济困难家庭、进城务工人员子女平等接受义务教育。加强义务教育学校标准化建设，整合教育资源，推动城乡义务教育协调均衡发展。进一步规范教育收费行为。深入实施中小学提高教育质量行动计划，推动素质教育发展。充分发挥职业教育作用，推广农村综合中学办学模式，加快培养适用型和技能型人才。重视学前教育，关心特殊教育，加快普及高中教育，搞好成人教育，大力发展高等教育，引导、规范、支持民办教育发展。深入实施人才强市战略，完善人才工作机制，留住高素质人才，引进急需人才，壮大人才队伍。推动文化大发展大繁荣。深化文化事业内部改革，增强文化活力。创新公共文化服务方式，保障人民群众的基本文化权益。完善市图书馆新馆建设，维修、改造市艺术剧院等文化场馆设施。抓好广播电视村村通、综合文化站建设、文化信息资源共享、农村电影放映、农家书屋五大工程。举办丰富多彩的纪念新中国成立60周年系列文化庆祝活动。大力发展文化产业，力争在新闻出版、广播影视、印刷发行、演艺娱乐、网络文化、工艺美术等方面实现新突破。打造体现地域文化特色和时代精神的文艺精品，推出一批有影响力的优秀剧目。继续完善《风刮卜奎》等一批优秀作品，推向东北乃至全国。以马戏为主体，推出一台常年演出的优秀节目。实施重大文化项目带动战略，推进鹤文化、民族区域文化、冰雪文化、红色文化、绿色生态文化等文化产业群建设。挖掘、整理和保护历史文化资源，搞好文物古建筑修缮工作。以网络文化管理为重点，加大文化市场监管力度。加快医疗卫生事业发展。积极争取资金，加快农村三级卫生服务网络和社区卫生服务中心建设。加强农村医护人员培养和监管，提高农村医疗卫生服务水平。进一步强化新型农村合作医疗管理。完善疾病防控体系和公共卫生服务体系。筹建中医大楼，推动中医药事业发展。扎实做好体育、人口计生等工作。以尼尔基斯湖滑雪场及现有体育场馆等为基地，促进全民体育运动开展。办好全市第八届体育运动大会，积极承办国内外大型体育赛事，大力扶持群众性体育运动、竞技体育运动和趣味体育运动开展。落实生育政策，稳定低生育水平，全面提高出生人口素质。抓好流动人口生育管理。深入实施计划生育家庭特别扶助制度和“生殖健康促进计划”。做好“双拥”和民兵预备役工作。加强民族宗教事务管理。搞好第二次全国经济普查工作。继续做好外事、侨务、人防、气象、防震减灾、档案、地方志等工作，支持妇女儿童、关心下一代和残疾人事业发展。我们这个历史文化厚重的城市，就是要建设一个公共产品更加丰富，社会服务更加优质，各项事业蓬勃发展的国家级历史文化名城。

　　（八）切实保障和改善民生。继续抓好大事实事，千方百计解决人民群众关心的热点、难点问题。扩大社会就业。认真贯彻实施就业促进法和劳动合同法，规范改善劳动关系，鼓励支持困难企业采取在岗培训、轮班工作等办法，尽量不裁员。统筹城乡就业和劳动力转移工作，发展中介服务组织，强化就业和创业培训。继续实施创业带就业工程，做好小额担保贷款发放工作，引导支持劳动者自主创业、灵活就业。积极开展“4050”人员、零就业家庭成员等困难群体的就业援助活动，大力开发公益性岗位，帮助困难群体就业再就业。力争实现下岗失业人员再就业7.5万人，其中实现困难人员就业1.2万人。做好大学生就业和退役军人安置工作。提高社会保障能力。继续推进户籍管理制度改革。完善城乡最低生活保障制度，做到应保尽保。切实管好用好社会保险基金，逐步做实养老保险个人账户。加大城镇居民基本医疗保险工作力度。扩大城镇职工基本养老、医疗、失业、工伤、生育保险覆盖面。做好贫困人口医疗救助和法律援助工作。发展社会福利和慈善事业，加强老龄、优抚安置、“五保”供养和扶贫解困工作。加大对革命老区贫困村的扶持力度。增设社区建设专项资金，适当提高社区工作人员生活补贴标准。开展城乡和谐社区创建活动，提高社区服务水平。完善村民自治制度，建立民主决策、民主管理的长效机制。加快保障性住房建设。健全廉租住房保障制度，建设廉租住房8210户，发放廉租住房补贴1.9万户、2900万元。大力推进“两改”工程，本着适度超前的原则，改造市区棚户区120万平方米，力争150万平方米，建设棚改回迁房140万平方米；改造县（市）棚户区50万平方米，建设棚改回迁房60万平方米。完成农村泥草房改造4.29万户。完善居民生活必需的配套设施建设，促进供水、供热、供气等公用事业健康发展。抓好安全生产。认真落实安全生产责任制，切实加大安全生产隐患排查、整改和监管力度，坚决防止重特大安全生产事故发生。继续开展食品、药品、日常生活用品质量安全专项整治活动，确保公共安全。搞好社会治安。深入开展“秋风行动”和“平安鹤城”创建活动，加强社会治安综合治理，完善城乡打防控一体化建设，严厉打击各种犯罪行为，努力创造安定有序的社会环境。认真解决信访问题。落实信访工作责任制和领导干部信访接待日、重点案件包案等制度。坚持依法治访，完善人民内部矛盾调处机制，加快案件办结速度，着力解决历史积案，严格控制群体访和越级访。加强应急管理。完善应急机制，普及应急知识，提高快速反应能力，有效预防和处置突发事件。我们就是要努力建设一个就业更加充分，保障更加完善，社会更加安定，学有所教，劳有所得，病有所医，老有所养，住有所居，人民群众更有安全感和幸福感的和谐城市。

　　（九）进一步加强政府自身建设。坚持以人为本、执政为民，着力提高行政效能，建设服务政府、责任政府、法治政府和廉洁政府。提高公务员队伍素质。大兴学习和调查研究之风，认真学习中国特色社会主义理论体系，钻研市场经济、现代金融等知识和国家各项政策，提高理论素养、文化修养和工作本领，创造性开展工作。认真落实国家有关公务员管理的各项规定，努力建设一支讲文明、守规矩、能攻坚、高素质的公务员队伍。依法规范行政。加强立法工作，深化行政执法体制改革，做好行政复议工作。健全专家咨询、决策评估、合法性审查、社会公示和听证、公开征求社会意见、反馈纠偏、责任追究等制度，推动决策的科学化、民主化。推进政务公开，提高政府工作透明度。严格按照制度、规则和程序办事。从治理“科所队底梗阻”现象入手，坚决纠正行业不正之风；加大诚信建设力度，努力营造重信守诺的经济环境。实行严格的行政问责制，依据有关规定，严肃查处擅自对企业检查、收费、罚款和在审批服务企业中吃拿卡要的单位及个人。加强督办检查，确保各项工作落到实处。加强廉政建设。遵守党风廉政建设的各项规定。加强制度建设，靠制度管人、管钱、管物，从源头上防止腐败发生。加强重点监督，确保重大项目、重要决策行政监察的全程及时跟进。厉行节约，把有限的资金和资源用在加快发展、改善民生上。强化审计监督检查。严厉查处各类违法违纪案件。深化行政管理体制改革。按照中央和省的统一部署，以转变职能为核心，积极稳妥地调整规范政府组织结构，因地因事设置机构。严格控制编制，向基层倾斜。推进政府社会管理职能创新，理顺城市管理体制。我们就是要建设一个更加开拓创新，更加勤勉尽责，更加团结务实，更加廉洁高效的人民满意的政府。

　　各位代表！展望鹤城美好的未来，令人鼓舞，催人奋进。我们的使命崇高而神圣，我们的责任重大而光荣。让我们更加紧密地团结起来，在市委的领导下，凝聚全市人民的智慧和力量，自力更生，艰苦奋斗，努力夺取全面建设小康社会的新胜利！
